# Robust Reference Powered Association Test of Genome-Wide Association Studies

**DOI:** 10.3389/fgene.2019.00319

**Published:** 2019-04-09

**Authors:** Yi Wang, Yi Li, Meng Hao, Xiaoyu Liu, Menghan Zhang, Jiucun Wang, Momiao Xiong, Yin Yao Shugart, Li Jin

**Affiliations:** ^1^Ministry of Education Key Laboratory of Contemporary Anthropology, Collaborative Innovation Center for Genetics and Development, School of Life Sciences, Shanghai, China; ^2^Human Phenome Institute, Fudan University, Shanghai, China; ^3^Institute of Sixth-Sector Industrialization Research, Fudan University, Shanghai, China; ^4^State Key Laboratory of Genetic Engineering, Collaborative Innovation Center for Genetics and Development, School of Life Sciences, Fudan University, Shanghai, China; ^5^Human Genetics Center, School of Public Health, University of Texas Houston Health Sciences Center, Houston, TX, United States

**Keywords:** GWAS, reference, public datasets, test statistic T, online tool

## Abstract

Genome-wide association studies (GWASs) have identified abundant genetic susceptibility loci, GWAS of small sample size are far less from meeting the previous expectations due to low statistical power and false positive results. Effective statistical methods are required to further improve the analyses of massive GWAS data. Here we presented a new statistic (Robust Reference Powered Association Test^1^) to use large public database (gnomad) as reference to reduce concern of potential population stratification. To evaluate the performance of this statistic for various situations, we simulated multiple sets of sample size and frequencies to compute statistical power. Furthermore, we applied our method to several real datasets (psoriasis genome-wide association datasets and schizophrenia genome-wide association dataset) to evaluate the performance. Careful analyses indicated that our newly developed statistic outperformed several previously developed GWAS applications. Importantly, this statistic is more robust than naive merging method in the presence of small control-reference differentiation, therefore likely to detect more association signals.

## Introduction

Genome-wide association studies (GWASs) have been widely applied with the goals to detect genetic variants which contribute to complex traits in the past decade (McCarthy et al., 2008). In general, allele frequencies of genetic variants are compared between cases that are supposed to have a high prevalence of susceptibility alleles and controls that are considered to have a lower prevalence of such alleles. And genomic loci correlated with various traits had been detected using the efficient approaches (He et al., 2009).

Although GWASs have led to abundant significant findings (Easton et al., 2007; Hakonarson et al., 2007; Parkes et al., 2007; Zeggini et al., 2007; Thomas et al., 2008), a few practical difficulties hinder the discovery of more rare or low-frequency genetic variants. For example, limited sample sizes make it difficult to achieve high statistical power which shows the probability of identifying the latent genetic variants (Wellcome Trust Case Control Consortium, 2007; He et al., 2009). Besides, difference in genetic background, also known as population stratification, between cases and controls could inflate type I error rate, thereby, leading to increasing level of false positive findings (Bacanu et al., 2000).

Availability of large public datasets contain large amount of useful genetic information. Utilizing large public datasets as reference may increase the sample size and ease the low power brought by insufficient sample size. However, population stratification and genotyping platform differences (He et al., 2009) between public datasets and cases may lead to inflated type I error rate. Our study aim is to appropriately utilize the public datasets to conservatively ameliorate the situation of small samples and low statistical power. In this manuscript, we describe a novel method to properly use public datasets (gnomad) as reference. In particular, we introduce a large public population as reference which has similar genetic background with control group. The null hypothesis was that allele frequency of SNPs among case (ca), control (co) and reference (re) was equal, and that the control and reference might compose a new large reference (co+re).

Specifically, we constructed a new test statistic T = ln(P(ca-(co+re)))-ln(P(co-re)) in which P(ca- (co + re)) is *p*-value of 2 × 2 Fisher exact test (Fisher, 1922, 1954; Agresti, 1992) of the SNP between case and control+reference, and P(co - re) is *p*-value of 2 × 2 Fisher exact test (Fisher, 1954; Agresti, 1992) of the SNP between control and reference. The T statistic takes differences between control and reference into account, which is more robust than P(ca - (co + re)) or P(ca - re)).

In this manuscript, we present a method to use public datasets as reference in the association analysis. The results of simulation and real data application showed that our new method could increase statistical power, particularly for small GWAS researches in real application. And the online tool (Robust Reference Powered Association Test^2^) has been made available.

## Results

### Results From Simulation Study

To evaluate the performance of our model for various situations, we simulated six parameters to compute the desired statistical power. The parameters include case sample size, allele frequency in case, control sample size, allele frequency in control, reference sample size and allele frequency in reference. Six different parameters were set to several typical values to simulate real scenarios. The case and control size were set from small to large. Also, allele frequencies were set from rare to common. Additionally, we selected two different reference of 10,000 and 100,000 samples. In detail, we set case size (case = 100, 500, 1000, 3000), control size (control = 0.5^∗^case, 1^∗^case, 2^∗^case, 5^∗^case), reference size (reference = 10,000, 100,000). And allele frequencies were set in the reference (ref = 0.001, 0.01, 0.05, 0.15, 0.3), in control (frequencies = 1^∗^ref, 1.1^∗^ref) and in case (frequencies = 1^∗^ref, 1.1^∗^ref, 1.5^∗^ref, 3^∗^ref). Totally, there were 128,000 different combinations (see Supplementary Table S1). Given sample size (N) and allele frequency (q), we simulated the count of allele which followed binomial distribution [B(2N, q)] for 100000 times. We drawn the QQ plot (Turner, 2014) of a representative simulation under H0 to assess the test statistic is well-calibrated (case size = 500, allele frequency in case = 0.165, control size = 500, allele frequency in control = 0.165, reference size = 10000, allele frequency in reference = 0.165) (Supplementary Figure S1). We have selected several representative cases to analyze the whole results of simulations (Table 1). First, supposed that the sample size of case, control and reference equaled 500, 500, and 10,000, respectively which were close to the real conditions. And allele frequencies in case, control, and reference were initialized to be common (0.15). As indicated in Table 1, the type 1 error rate of T was a bit higher than that of ca-(co+re), while both were less than significant levels (alpha = 0.05, 0.01, 0.001). When there was small population genetic differentiation between control and reference, allele frequencies in case and control equaled 0.165 while allele frequency in reference was set as 0.15. In this case, the type 1 error rate of T was less than that of ca-(co+re). Therefore, as mentioned in Section “Materials and Methods,” our new test is more robust than the simple P(ca - (co + re)) method in the presence of small control-reference differentiation.

**Table 1:** Simulation power in representative cases.

Groups	*N*	Frequency	Alpha	ca-(re+co)	co - re	ca - co	T
Case	500	0.150	0.050	0.0472	0.0463	0.0431	0.0481
Control	500	0.150	0.010	0.0092	0.0089	0.0079	0.0094
Ref	10000	0.150	0.001	0.0008	0.0008	0.0008	0.0008
Case	500	0.165	0.050	0.2290	0.2505	0.0426	0.1465
Control	500	0.165	0.010	0.0895	0.0998	0.0080	0.0568
Ref	10000	0.150	0.001	0.0193	0.0223	0.0008	0.0118
Case	500	0.165	0.050	0.2142	0.4228	0.0478	0.0979
Control	1000	0.165	0.010	0.0807	0.2098	0.0094	0.0359
Ref	10000	0.150	0.001	0.0182	0.0655	0.0009	0.0078
Case	500	0.165	0.050	0.1771	0.7398	0.0485	0.0323
Control	2500	0.165	0.010	0.0622	0.5127	0.0097	0.0109
Ref	10000	0.150	0.001	0.0124	0.2509	0.0009	0.0021
Case	500	0.165	0.050	0.2496	0.0464	0.1367	0.2383
Control	500	0.150	0.010	0.0992	0.0091	0.0429	0.0949
Ref	10000	0.150	0.001	0.0228	0.0009	0.0075	0.0215
Case	500	0.225	0.050	1.0000	0.0465	0.9891	0.9998
Control	500	0.150	0.010	0.9997	0.0092	0.9524	0.9990
Ref	10000	0.150	0.001	0.9965	0.0009	0.8300	0.9941
Case	500	0.030	0.050	0.9953	0.0418	0.8799	0.9919
Control	500	0.010	0.010	0.9808	0.0076	0.7038	0.9742
Ref	10000	0.010	0.001	0.9259	0.0008	0.4246	0.9152

As shown in Table 1, false positive results might occur if there was small population genetic differentiation between control and reference while no differentiation between case and control. To evaluate the influence of control size, we simulated different control sizes with the aim to detect the false positive rate under different allele frequencies of case and control (see Supplementary Table S2). We found that the false positive rate of P(T) would decrease when control size was augmented. Furthermore, P(T)’s type 1 error rate was less than P(ca - co)’s when the sample size of the controls was five times larger than that of the cases. The false positive results were kept as low as possible when the sample size of the controls was four times larger than that of the cases (see Supplementary Table S2). In addition to population stratification between control and reference, allele frequency differentiation between case and control, between case and reference due to different genetic background were also simulated (see Supplementary Table S3). When there was population stratification between case and control without differentiation between case and reference, the type 1 error rate of T was less than significant levels (alpha = 0.05, 0.01, 0.001) while the type 1 error rate of ca-(co+re) and ca-co were large than significant levels. In another scenario of population stratification between case and reference without differentiation between case and control, results of T might be false positive. However increased sample size would control the type 1 error rate.

When the allele frequency in case was different from that in control, T’s power was always higher than power of ca-co. We could find that when there was a slight change of allele frequency in case, the power of T was much higher than power of ca-co, indicating that our method had high sensitivity for GWAS. When the allele frequency in case was much higher than control’s, the power approached to 1 with remarkable increase of T’s power. When the allele frequency was rare, we could also draw the same conclusion that our method could keep false positive rate low and drastically increase the statistical power.

### Results From the Psoriasis GWAS Datasets

We applied our newly developed method to two psoriasis GWAS (Nair et al., 2006; Fang et al., 2011) datasets to evaluate the performance. 1,590 subjects (915 cases, 675 controls) in the general research use (GRU) group and 1,133 subjects (431 cases and 702 controls) in the autoimmune disease only (ADO) group were analyzed.

For the GRU group and ADO group, SNPs that failed to pass the HWE exact test were filtered (we used the *p* = 0.001 as the threshold). Fisher exact test was used to compute the *p*-value of allele frequency for each SNP. The threshold of *p*-value was set as 1.2 × 10^-7^ by Bonferroni Correction. Then we selected first 100 SNPs of lowest *p*-values for further analysis. Two different large public datasets, gnomad.genome. NFE (Non-Finnish European, *N* = 7,509) (ref1) (Lek et al., 2016) and gnomad.exome.NFE (Non-Finnish European, *N* = 55,860) (ref2) (Lek et al., 2016), were selected as the reference groups. So there were four combinations: GRU group vs. ref1 (GRU_ref1), GRU group vs. ref2 (GRU_ref2), ADO group vs. ref1 (ADO_ref1) and ADO group vs. ref2 (ADO_ref2). For each condition, we computed the *p*-value of our model (see Supplementary Table S4). Specially for the exome dataset (ref2), some SNPs not in the exome were excluded from the table.

**FIGURE 1:**
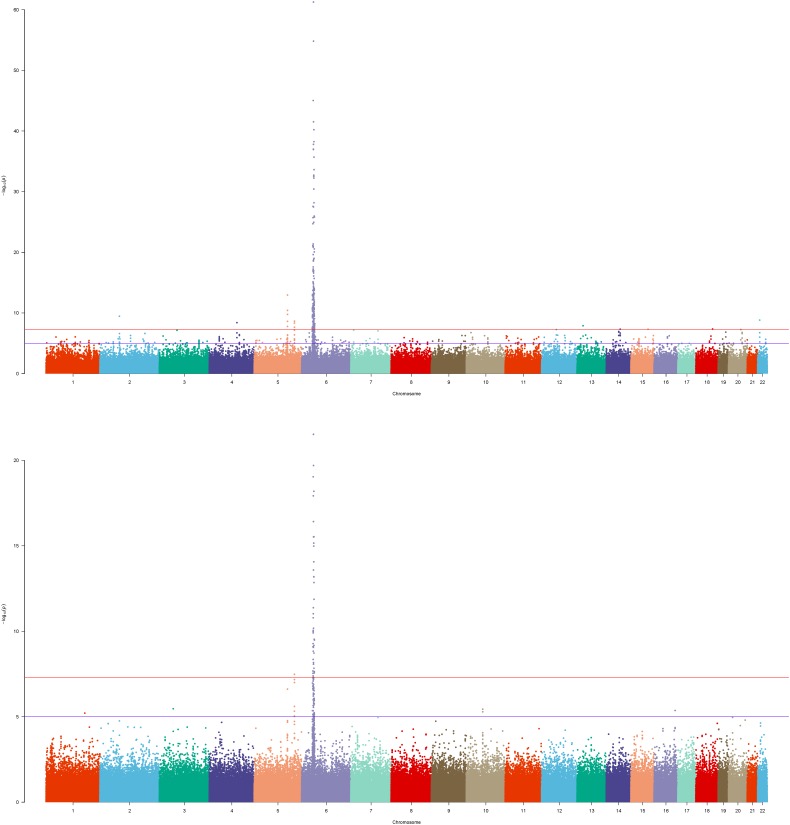
Manhattan plot of GRU group of Psoriasis GWAS datasets. The bottom figure corresponded to the P(ca-co) and top figure corresponded to the P(T), and reference was ref1.

**FIGURE 2:**
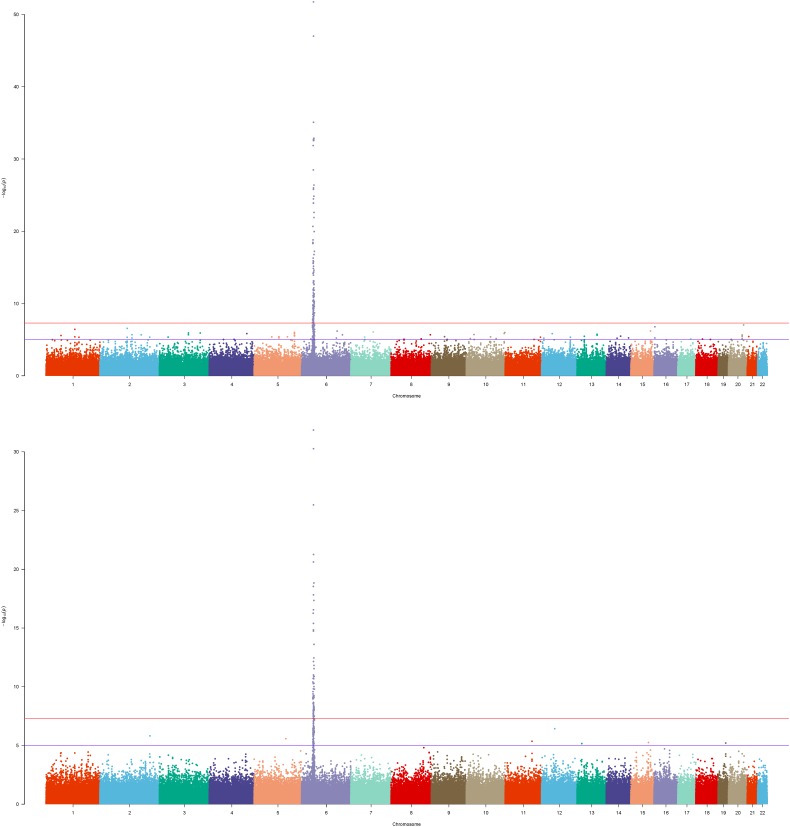
Manhattan plot of ADO group of Psoriasis GWAS datasets. The bottom figure corresponded to the P(ca-co) and top figure corresponded to the P(T), and reference was ref1.

To inspect the improvement of *p*-values in the whole level, we drew the Manhattan plot of GRU group (Figure 1) and ADO group (Figure 2) respectively. We observed notable changes of *p*-values before and after performing our method. The peaks of Figure 1, 2 jumped from about 1e-22 and 1e-32 to 1e-62 and 1e-52 respectively. Also the amount of SNPs with *p*-value between 1e-5 and 1e-8 has increased. The positive SNPs (*rs12191877*, *rs9468933*, etc.) became more positive due to the added genetic information of reference. Also, our method rescued a few SNPs which turned from negative to positive (see Supplementary Table S5). Then, we searched pubmed literature and found some SNPs associated with psoriasis that had been reported by other studies. Moreover we checked these novel significant SNPs in GWAS catalog (Macarthur et al., 2016) to validate whether the associations were replicated in recent related large GWAS studies. By integrating the results of ref1 and ref2, we found that the SNPs with ref2 as reference were included in the results of ref1. And we presented the novel SNPs of GRU group (Table 2) and ADO group (Table 3). We calculated the genomic inflation factor, also known as lambda (λ). In GRU group, λ of P(T) and P(ca - co) were 1.08 and 0.98 respectively. And in ADO group, λ of P(T) and P(ca - co) were 0.81 and 0.96, respectively. Besides QQ plot of P(T) and P(ca - co)were drawn to inspect the distributions (Supplementary Figure S2).

**Table 2:** SNPs rescued from negative to positive of GRU group.

RS	P(co-re)	P(ca-co)	P(T)	RS	P(co-re)	P(ca-co)	P(T)
rs2021723	0.0943555	1.66E-07	7.99E-22	rs9266825	0.796691	2.03E-07	1.25E-13
rs1015465	0.0777779	2.22E-07	9.45E-22	rs9266845	0.747484	2.42E-07	2.50E-13
rs6906662	0.217811	3.06E-07	9.88E-20	rs9295991	0.747859	2.54E-07	3.28E-13
rs3094205	0.181069	1.76E-07	2.35E-19	rs9266813	0.974968	1.37E-06	3.87E-13
rs9262498	0.0562976	2.28E-06	2.62E-18	rs9266846	0.699231	2.37E-07	4.00E-13
rs9262492	0.0782655	2.24E-06	5.84E-18	***rs1051792***	0.949952	1.67E-06	4.77E-13
rs9295924	0.198905	1.04E-06	1.01E-17	rs176095	0.945133	2.04E-06	1.94E-12
rs6933779	0.225183	8.90E-07	1.15E-17	rs2239518	0.443697	1.48E-07	3.18E-12
rs2395471	0.419017	1.28E-07	1.37E-17	rs3130685	0.587326	5.00E-07	1.34E-11
rs10947208	0.470313	1.86E-07	2.65E-17	rs2894176	0.562234	6.25E-07	2.18E-11
***rs13437088***	0.534501	2.40E-07	8.09E-17	***rs2442719***	0.626544	1.07E-06	4.47E-11
rs4711229	0.663943	2.74E-07	7.27E-16	rs2734573	0.408163	4.93E-07	1.56E-10
rs2853950	0.863224	1.30E-07	1.70E-15	rs2858332	0.495284	1.11E-06	2.20E-10
***rs7192***	0.543898	8.41E-07	8.66E-15	rs3130048	0.156454	2.13E-07	2.73E-09
rs7194	0.563064	1.02E-06	1.25E-14	rs1003879	0.177049	4.68E-07	7.88E-09
rs12203586	0.269737	7.01E-06	1.27E-14	rs9391858	0.365437	6.00E-06	1.34E-08
rs2856726	0.309997	5.83E-06	1.90E-14	rs6894567	0.300402	4.80E-06	6.88E-08
***rs20541***	1	2.44E-07	1.13E-13				

*The SNPs reported in pubmed were shown in bold and italics.*

**Table 3:** SNPs rescued from negative to positive of ADO group.

RS	P(co-re)	P(ca-co)	P(T)	RS	P(co-re)	P(ca-co)	P(T)
rs3095250	0.322996	1.94E-07	1.33E-12	rs3130043	0.6013	3.96E-07	3.30E-09
rs3095254	0.261656	2.77E-07	1.63E-12	rs1265762	0.498308	2.73E-07	4.85E-09
rs9468937	0.4478	4.06E-07	8.58E-12	rs2074504	0.551801	3.83E-07	6.91E-09
rs3094214	0.801659	1.38E-07	1.29E-11	***rs3130573***	0.413724	3.83E-07	1.15E-08
rs7772549	0.647633	5.40E-07	3.28E-11	rs6927461	0.527086	5.62E-07	1.45E-08
rs2524222	0.972433	5.09E-07	1.60E-10	rs2239518	0.328123	4.36E-07	2.12E-08
rs2524082	0.692881	1.29E-07	3.85E-10	rs2844724	0.397193	3.59E-07	2.32E-08
rs2523857	0.775127	4.12E-07	9.95E-10	rs16899213	0.209543	1.39E-07	6.49E-08

*The SNPs reported in pubmed was shown in bold and italics.*

For GRU group, *rs13437088* [P(T) = 8.09E-17)], located 30 kb centromeric of *HLA-B* and 16 kb telomeric of *MICA* (MIM: 600169), had been previously reported to be associated with psoriasis (Feng et al., 2009). Besides, *rs7192* [P(T) = 8.66E-15] and *rs20541* [P(T) = 1.13E-13] were candidate causal SNPs at leukocyte antigen (HLA) loci (MIM: 142395) which played an important roles in pathways of psoriasis (Lee et al., 2012). Also, *rs1051792* [P(T) = 4.77E-13] in the *MICA* gene (rs1051792) had also been suggested to be specific for purely cutaneous manifestations of psoriasis (Bowes et al., 2015). And SNP *rs2442719* [P(T) = 4.47E-11], located only 1 kb from the telomeric end of *HLA-B* (MIM: 142830), also exhibited significant association with psoriasis (Feng et al., 2009). For the ADO group, *rs3130573* [P(T) = 1.70E-10] was located in *PSORS1C1* (MIM: 613525) gene which was a major susceptibility locus for psoriasis (Fan et al., 2008). Likewise we drawn the QQ plot of P(T) and P(ca - co) (Supplementary Figure S3).

### Results From the Schizophrenia GWAS Datasets

We also applied our newly developed method to one schizophrenia GWAS (Zuo et al., 2013) dataset to evaluate the performance. The SNPs that did not pass the HWE exact test were filtered (*p* = 0.001). Fisher exact test was used to compute the *p*-value of allele frequency for each SNP. There was no statistically significant SNP with the threshold as 7.14 × 10^-8^ by Bonferroni Correction. The large public datasets, gnomad.genome.NFE (Non-Finnish European, *N* = 7509) (ref1) (Lek et al., 2016) was selected as reference to compute the *p*-value of our model (see Supplementary Table S6).

**FIGURE 3:**
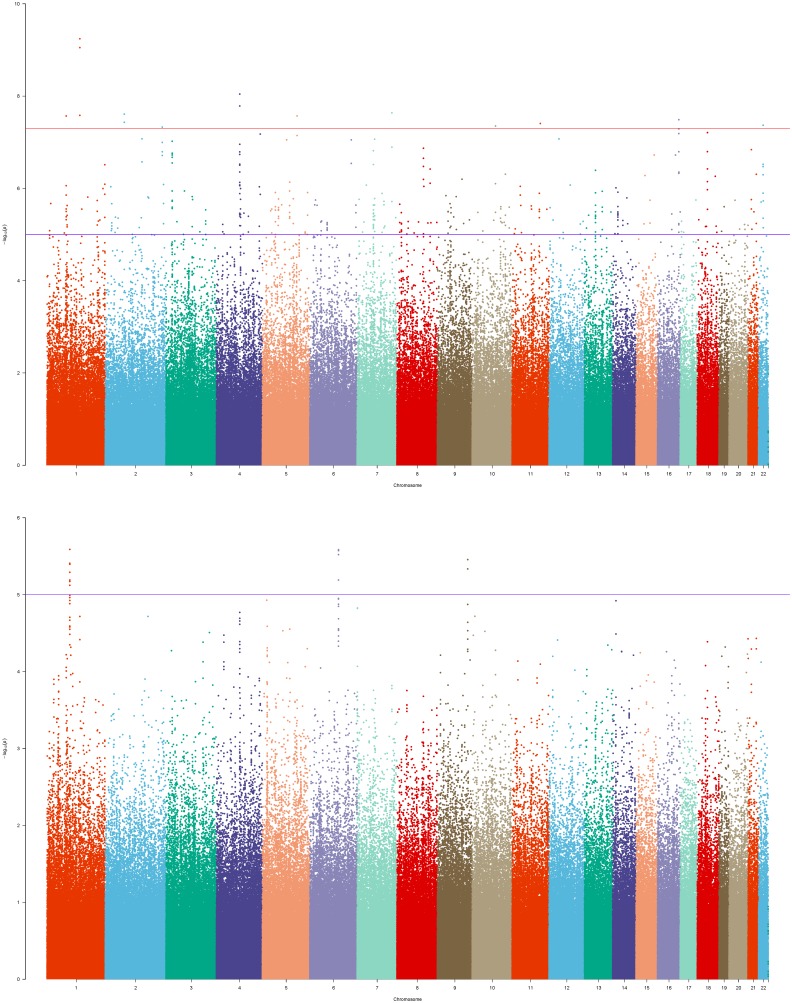
Manhattan plot of schizophrenia GWAS dataset. The bottom figure corresponded to the P(ca-co) and top figure corresponded to the P(T), and reference was ref1.

For the schizophrenia GWAS dataset (Zuo et al., 2013), the typical Fisher exact test did not yield genome-wide significant findings. After the introduction of reference, we found that several novel SNPs were associated with schizophrenia (Table 4). The Manhattan plot (Figure 3) clearly showed that before performing our method there were only a few SNPs with *p*-value below 1e-5 in the bottom figure. And after performing our method, plenty of SNPs came to the fore with *p*-value between 1e-5 and 1e-8. In addition, there were significant SNPs with *p*-value below 7e-8, some of which had been reported in previous studies. The SNP *rs12140791* is located in *NOS1AP* (MIM: 605551) gene which is essential for brain development and function and of potential relevance to schizophrenia (Glessner et al., 2010). The *rs17021364* and *rs110974077* were reported to be associated with schizophrenia in a genome-wide meta-analysis (Wang et al., 2010). The *rs35648* (*p*-value = 9.65E-5) was also reported by a previous large-scale GWAS (Shi et al., 2009). The genomic inflation factors were 0.77 and 1.01 for P(T) and P(ca-co) respectively. Also QQ plot of P(T) and P(ca - co) were drawn (Supplementary Figure S4).

**Table 4:** SNPs rescued from negative to positive of schizophrenia dataset.

RS	P(co-re)	P(ca-co)	P(T)	RS	P(co-re)	P(ca-co)	P(T)
***rs12140791***	0.497902	1.92E-05	5.73E-10	***rs17021364***	0.624051	5.04E-05	1.64E-08
rs10753758	0.399987	3.84E-05	8.91E-10	***rs35648***	0.677469	5.29E-05	4.46E-08
***rs1109740777777***	0.814451	2.03E-05	9.06E-09				

*The SNPs reported in pubmed was shown in bold and italics.*

## Materials and Methods

### Framework of Robust Reference Powered Association Test

Suppose we have three populations: case, control and public data pools, intuitively we want to merge the control and reference population to form a large control pool to gain more power on allele-disease association test. However, we are concerned about the potential population differentiation and genotyping platform difference between control and the reference (He et al., 2008).

A simplistic way to alleviate such concern is to perform a control (co) vs. reference (re) Fisher exact test (Fisher, 1922). If a *p*-value is not significant and the control sample size is not too small, then this concern is resolved. However, choosing the significance level is arbitrary and the decision is subjective. We need an objective version of such procedure to correct the effect of co-re (control vs. reference) difference.

**FIGURE 4:**
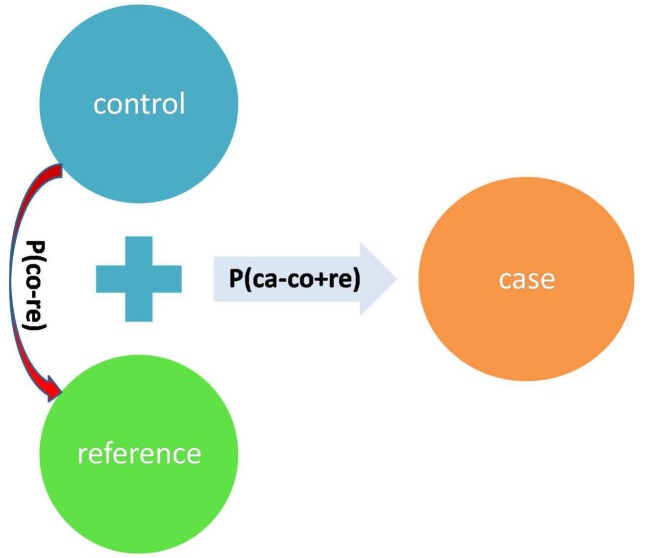
The summarized design of the test statistic T.

Denote the *p*-value of 2 × 2 Fisher exact test (Fisher, 1922, 1954; Agresti, 1992) of the SNP between case and control+reference as P(ca - (co + re)). Denote the *p*-value of 2 × 2 Fisher exact test of the SNP between control and reference as P(co - re). We define a test statistic: T = ln(P(ca - (co + re))) - ln(P(co - re)) (Figure 4). T will be smaller if the difference between case and control + reference is larger, while T will be larger if the difference between control and reference is larger. Therefore, T is a more robust statistic than P(ca - (co + re)) as it takes the control-reference difference into account by penalizing P(co - re).

Our null hypothesis is that the allele frequency of the SNP is equal among cases, controls and references. Under the null hypothesis, both P(ca - (co + re)) and P(co - re) are independent and uniformly distributed on (0, 1). Under the null hypothesis, -ln(P(ca - (co + re))) and -ln(P(ca - co)) are exponentially distributed with parameter 1 (Casella and Berger, 2002). And T is the difference of two exponentially distributed variables, thus T is Laplace distributed as T∼ Laplace (0, 1) (Mcneil, 2003). The one side *p*-value of T is as follow:

$$P(T)=\left\{\frac{\frac{P(ca-(co+re))}{2P(co-re)},P(ca-(co+re))\leq P(co-re)}{\frac{1-P(co-re)}{2P(ca-(co+re))},P(ca-(co+re))>P(co-re)}\right\}$$

From the above formula one can easily see that the P(co - re) acts as a penalizer/normalizer against P(ca- (co + re)). Therefore, our new statistic T is more robust than the simple P(ca - (co + re)) method in the presence of small control-reference differentiation. However, in the presence of strong population differentiation or genotyping platform difference, even our correction may not be effective, we therefore need to restrict P(co - re) to be not significant with a suggested threshold of 0.01.

### The Psoriasis GWAS Datasets

We obtained the psoriasis datasets (Nair et al., 2006; Fang et al., 2011), as a part of the Collaborative Association Study of Psoriasis (CASP), from the Genetic Association Information Network (GAIN) database (dbGaP Accession No. phs000019.v1. p1), a partnership of the Foundation for the National Institutes of Health. All genotypes were filtered by checking for data quality (Feng et al., 2009). A dermatologist diagnosed all psoriasis (MIM: 177900) cases. Each participant’s DNA was genotyped with the Perlegen 500K array. Both cases and controls agreed to sign the consent contract, and controls (≥18 years old) had no confounding factors relative to a known diagnosis of psoriasis.

### The Schizophrenia GWAS Dataset

The schizophrenia dataset (Zuo et al., 2013) came from the GAIN dataset (dbGaP Access No. phs000021.v1.p1), including 1,195 cases with schizophrenia (MIM: 181500) and 954 controls. The subjects were genotyped on AFFYMETRIX AFFY_6.0 platform. All subjects were at least 18 years old. The cases included 746 males (41.9 ± 10.8 years) and 449 females (43.0 ± 9.8 years); and the controls included 362 males (46.2 ± 13.7 years) and 592 females (45.0 ± 12.9 years). Affected subjects met lifetime DSM-IV criteria for schizophrenia (American Psychiatric Association 1994). Cases were excluded if they had worse than mild mental retardation, or if their psychotic illness was judged to be secondary to substance use or neurological disorders. Controls were excluded if they did not deny all of the following psychosis screening questions: treatment for or diagnosis of schizophrenia or schizoaffective disorder; treatment for or diagnosis of bipolar disorder or manic-depression; treatment for or diagnosis of psychotic symptoms such as auditory hallucinations or persecutory delusions.

## Discussion

Associations between SNPs and complex traits were detected by comparing frequencies of alleles in case and control group in GWAS (McCarthy et al., 2008). Several significant SNPs have been identified in classic GWAS studies (Duerr et al., 2006; Hunter, 2007; Hunter et al., 2007; Scott et al., 2007). However, other SNPs of low frequencies which contribute to the complex traits remain hidden in the false negative results (Frayling et al., 2007; Willer et al., 2008; Sanna et al., 2008; Nair et al., 2009; Wang et al., 2013). To identify more novel susceptibility loci, large-scale GWAS is a costly and time-consuming approach. It is necessary to design more sensitive and accurate statistical methods. Interestingly, the results indicated that our method could increase the power which may contribute to detecting more significant SNPs.

Our simulations focused on several representative cases to evaluate the performance of the new model. When the allele frequencies of case, control and reference were equal, the power of T was higher than power of ca-(co+re), while both less than false positive rate. False positive results would be produced in the presence of small population genetic differentiation between control and reference. However, as the control size increased, the false positive rate of P(T) would be reduced (Table 1). The false positive rate would be controlled well, when the control sample was large enough. What’s more, T’s power was always higher than power of ca-co, when the allele frequencies of case and control were different, indicating that our method was more robust and had high sensitivity for GWAS.

Considering that allele frequency divergence of multiple variants due to different platforms is more serious than slight population stratification in practical application, we designed the statistic based on single variant rather than multiple variants. Allowing slight population stratification between control and reference, the simulation has shown the large sample size of control would suppress the false positive results. Moreover the statistical power and utility of our method were also elevated.

For the psoriasis GWAS datasets (Nair et al., 2006; Fang et al., 2011), the positive SNPs became more positive and some of the negative SNPs turned to be positive after application of our method. The rescued SNPs, *rs1343708* (Feng et al., 2009), *rs7192* (Lee et al., 2012), *rs20541* (Lee et al., 2012), *rs1051792* (Bowes et al., 2015), *rs2442719* (Feng et al., 2009), and *rs3130573* (Fan et al., 2008) were identified to be true positive. For the schizophrenia GWAS dataset (Zuo et al., 2013), typical Fisher Exact tests produced no significant positive genetic loci. However, our method found that SNPs *rs12140791* (Glessner et al., 2010), *rs10753758*, *rs11097407* (Wang et al., 2010), *rs17021364* (Wang et al., 2010), *rs35648* (Shi et al., 2009) could potentially be associated with schizophrenia. The results indicated that our method could be sensitive to generate more positive SNPs.

In the aforementioned application of our method to the psoriasis GWAS genetic datasets (Nair et al., 2006; Fang et al., 2011), we selected two large public datasets as reference groups (Lek et al., 2016). With different references, the model will compute different *p*-values for the test statistic. In the case of scenario, the *p*-value is lower than threshold of 0.01, indicating significant differentiation between control and reference, the result would be false positive. In this situation, selecting an appropriate dataset as the reference is the key to obtain better result. In addition, selecting multiple different references is feasible with the online tool. By comparing and integrating the output of different references, reasonable significant P(T) could provide more effective information for SNPs associated with the traits.

Novel SNPs with weak positive signals could be discovered when the sample size of case and control are insufficient in GWAS (He et al., 2009). With the support of reference database, the new *p*-value of some false negative genetic loci would decrease significantly down to the threshold. And our test statistic T is more robust than P(ca - (co + re)) as it takes the control-reference difference into account by penalization of P(co - re).

We presented a new statistic T to use large public database as reference to reduce concern of potential population stratification. And the new statistic proposed here is effective to discover novel genome-wide significant loci with both small and large sample sizes.

## Author Contributions

YW conceived the idea and developed the software. YL, MH, and XL contributed data analysis, generating tables and figures, and manuscript writing. YW, YL, MH, XL, YS, and LJ contributed the theoretical analysis and manuscript revision. MX helped support the GWAS datasets. YW, YL, MH, XL, MZ, JW, and MX contributed to scientific discussion. All authors contributed to final revision of the manuscript.

## Conflict of Interest Statement

The authors declare that the research was conducted in the absence of any commercial or financial relationships that could be construed as a potential conflict of interest.

## Footnotes

2 http://drwang.top/gwas.html

## Supplementary Material

FIGURE S1 QQ plot under H0.

FIGURE S2 QQ plot of P(T) and P(Ca-Co) of GRU Group.

FIGURE S3 QQ plot of P(T) and P(Ca-Co) of ADO Group.

FIGURE S4 QQ plot of P(T) and P(Ca-Co) of Schizophrenia.

TABLE S1 128000 different combinations of six parameters in simulation study.

TABLE S2 Evaluation of the influence of control size in different model.

TABLE S3 Evaluation of the population stratification between case-reference.

TABLE S4 *p*-Value of different group combination for psoriasis datasets.

TABLE S5 Rescued SNPs by robust reference powered association test.

TABLE S6 *p*-Value of schizophrenia dataset.

## References

[B1] Agresti A. (1992). A survey of exact inference for contingency tables. *Stat. Sci.* 7 131–153. 10.1214/ss/1177011454

[B2] Bacanu S. A. Devlin B. Roeder K. (2000). The power of genomic control. *Am. J. Hum. Genet.* 66 1933–1944. 10.1086/302929 10801388 PMC1378064

[B3] Bowes J. Budu-Aggrey A. Huffmeier U. Uebe S. Steel K. Hebert H. L. (2015). Dense genotyping of immune-related susceptibility loci reveals new insights into the genetics of psoriatic arthritis. *Nat. Commun.* 6:6046. 10.1038/ncomms7046 25651891 PMC4327416

[B4] Casella G. Berger R. L. (2002). *Statistical Inference.* Boston, MA: Thomson Learning.

[B5] Duerr R. H. Taylor K. D. Brant S. R. Rioux J. D. Silverberg M. S. Daly M. J. (2006). A genome-wide association study identifies IL23R as an inflammatory bowel disease gene. *Science* 314 1461–1463. 10.1126/science.1135245 17068223 PMC4410764

[B6] Easton D. F. Pooley K. A. Dunning A. M. Pharoah P. D. Thompson D. Ballinger D. G. (2007). Genome-wide association study identifies novel breast cancer susceptibility loci. *Nature* 447 1087–1093. 10.1038/nature05887 17529967 PMC2714974

[B7] Fan X. Yang S. Huang W. Wang Z. M. Sun L. D. Liang Y. H. (2008). Fine mapping of the psoriasis susceptibility locus PSORS1 supports HLA-C as the susceptibility gene in the Han Chinese population. *PLoS Genet.* 4:e1000038. 10.1371/journal.pgen.1000038 18369457 PMC2265413

[B8] Fang S. Fang X. Xiong M. (2011). Psoriasis prediction from genome-wide SNP profiles. *BMC Dermatol.* 11:1. 10.1186/1471-5945-11-1 21214922 PMC3022824

[B9] Feng B. J. Sun L. D. Soltaniarabshahi R. Bowcock A. M. Nair R. P. (2009). Multiple loci within the major histocompatibility complex confer risk of psoriasis. *PLoS Genet.* 5:e1000606. 10.1371/journal.pgen.1000606 19680446 PMC2718700

[B10] Fisher R. A. (1922). On the interpretation of χ^2^ from contingency tables, and the calculation of P. *J. R. Stat. Soc.* 85 87–94. 10.2307/2340521

[B11] Fisher R. A. (1954). *Statistical Methods for Research Workers.* Edinburgh: Oliver and Boyd.

[B12] Frayling T. M. Timpson N. J. Weedon M. N. Zeggini E. Freathy R. M. Lindgren C. M. (2007). A common variant in the FTO gene is associated with body mass index and predisposes to childhood and adult obesity. *Science* 316 889–894. 10.1126/science.1141634 17434869 PMC2646098

[B13] Glessner J. T. Reilly M. P. Kim C. E. Takahashi N. Albano A. Hou C. (2010). Strong synaptic transmission impact by copy number variations in schizophrenia. *Proc. Natl. Acad. Sci. U.S.A.* 107 10584–10589. 10.1073/pnas.1000274107 20489179 PMC2890845

[B14] Hakonarson H. Grant S. F. Bradfield J. P. Marchand L. Kim C. E. Glessner J. T. (2007). A genome-wide association study identifies KIAA0350 as a type 1 diabetes gene. *Nature* 448 591–594. 10.1038/nature06010 17632545

[B15] He Y. Jiang R. Fu W. Bergen A. W. Swan G. E. Jin L. (2008). Correlation of population parameters leading to power differences in association studies with population stratification. *Ann. Hum. Genet.* 72 801–811. 10.1111/j.1469-1809.2008.00465.x 18652602 PMC2574891

[B16] He Y. Xu S. Jia C. Jin L. (2009). A design of multi-source samples as a shared control for association studies in genetically stratified populations. *Cell Res.* 19 913–915. 10.1038/cr.2009.75 19546887

[B17] Hunter D. J. Kraft P. Jacobs K. B. Cox D. G. Yeager M. Hankinson S. E. (2007). A common allele on chromosome 9 associated with coronary heart disease. *Science* 316 1488–1491. 10.1126/science.1142447 17478681 PMC2711874

[B18] Hunter D. J. E. A. (2007). A genome-wide association study identifies alleles in FGFR2 associated with risk of sporadic postmenopausal breast cancer. *Nat. Genet.* 39 870–874. 10.1038/ng2075 17529973 PMC3493132

[B19] Lee Y. H. Choi S. J. Ji J. D. Song G. G. (2012). Genome-wide pathway analysis of a genome-wide association study on psoriasis and Behcet’s disease. *Mol. Biol. Rep.* 39 5953–5959. 10.1007/s11033-011-1407-9 22201026

[B20] Lek M. Karczewski K. J. Minikel E. V. Samocha K. E. Banks E. Fennell T. (2016). Analysis of protein-coding genetic variation in 60,706 humans. *Nature* 536 285–291. 10.1038/nature19057 27535533 PMC5018207

[B21] Macarthur J. Bowler E. Cerezo M. Gil L. Hall P. Hastings E. (2016). The new NHGRI-EBI Catalog of published genome-wide association studies (GWAS Catalog). *Nucleic Acids Res.* 45 D896–D901. 10.1093/nar/gkw1133 27899670 PMC5210590

[B22] McCarthy M. I. Abecasis G. R. Cardon L. R. Goldstein D. B. Little J. Ioannidis J. P. (2008). Genome-wide association studies for complex traits: consensus, uncertainty and challenges. *Nat. Rev. Genet.* 9 356–369. 10.1038/nrg2344 18398418

[B23] Mcneil A. J. (2003). The laplace distribution and generalizations: a revisit with applications to communications, economics, engineering, and finance. *J. R. Stat. Soc.* 52 698–699. 10.1046/j.1467-9884.2003.t01-12-00383_14.x

[B24] Nair R. P. Duffin K. C. Helms C. Ding J. Stuart P. E. Goldgar D. (2009). Genome-wide scan reveals association of psoriasis with IL-23 and NF-κB pathways. *Nat. Genet.* 41 199–204. 10.1038/ng.311 19169254 PMC2745122

[B25] Nair R. P. Stuart P. E. Nistor I. Hiremagalore R. Chia N. V. Jenisch S. (2006). Sequence and haplotype analysis supports HLA-C as the psoriasis susceptibility 1 gene. *Am. J. Hum. Genet.* 75 827–851. 10.1086/503821 16642438 PMC1474031

[B26] Parkes M. Barrett J. C. Prescott N. J. Tremelling M. Anderson C. A. Fisher S. A. (2007). Sequence variants in the autophagy gene IRGM and multiple other replicating loci contribute to Crohn’s disease susceptibility. *Nature* 39 830–832. 10.1038/ng2061 PMC2628541 17554261

[B27] Sanna S. Jackson A. U. Nagaraja R. Willer C. J. Chen W. M. Bonnycastle L. L. (2008). Common variants in the GDF5-UQCC region are associated with variation in human height. *Nat. Genet.* 40 198–203. 10.1038/ng.74 18193045 PMC2914680

[B28] Scott L. J. Mohlke K. L. Bonnycastle L. L. Willer C. J. Li Y. Duren W. L. (2007). A genome-wide association study of type 2 diabetes in Finns detects multiple susceptibility variants. *Science* 316 1341–1345. 10.1126/science.1142382 17463248 PMC3214617

[B29] Shi J. Levinson D. F. Duan J. Sanders A. R. Zheng Y. Pe’er I. (2009). Common variants on chromosome 6p22.1 are associated with *schizophrenia*. *Nature* 460 753–757. 10.1038/nature08192 19571809 PMC2775422

[B30] Thomas G. Jacobs K. B. Yeager M. Kraft P. Wacholder S. Orr N. (2008). Multiple loci identified in a genome wide association study of prostate cancer. *Nat. Genet.* 40 310–315. 10.1038/ng.91 18264096

[B31] Turner S. D. (2014). qqman: an R package for visualizing GWAS results using Q-Q and manhattan plots. *bioRxiv* [Preprint]. 10.1101/005165

[B32] Wang K. S. Liu X. F. Aragam N. (2010). A genome-wide meta-analysis identifies novel loci associated with schizophrenia and bipolar disorder. *Schizophr. Res.* 124 192–199. 10.1016/j.schres.2010.09.002 20889312

[B33] Wang W. J. Yin X. Y. Zuo X. B. Cheng H. Du W. D. Zhang F. Y. (2013). Gene–gene interactions in IL23/Th17 pathway contribute to psoriasis susceptibility in Chinese Han population. *J. Eur. Acad. Dermatol.* 27 1156–1162. 10.1111/j.1468-3083.2012.04683.x 22909235

[B34] Wellcome Trust Case Control Consortium. (2007). Genome-wide association study of 14,000 cases of seven common diseases and 3,000 shared controls. *Nature* 447 661–678. 10.1038/nature05911 17554300 PMC2719288

[B35] Willer C. J. Sanna S. Jackson A. U. Scuteri A. Bonnycastle L. L. Clarke R. (2008). Newly identified loci that influence lipid concentrations and risk of coronary artery disease. *Nat. Genet.* 40 161–169. 10.1038/ng.76 18193043 PMC5206900

[B36] Zeggini E. Weedon M. N. Lindgren C. M. Frayling T. M. Elliott K. S. Lango H. (2007). Replication of genome-wide association signals in UK samples reveals risk loci for type 2 diabetes. *Science* 316 1336–1341. 10.1126/science.1142364 17463249 PMC3772310

[B37] Zuo L. Wang K. Zhang X. Y. Pan X. Wang G. Tan Y. (2013). Association between common alcohol dehydrogenase gene (ADH) variants and schizophrenia and autism. *Hum. Genet.* 132 735–743. 10.1007/s00439-013-1277-4 23468174 PMC3683370

