# Supplementary figures and images for: Robust Reference Powered Association Test of Genome-Wide Association Studies
Source: Front Genet. 2019 Apr 9;10:319. doi: 10.3389/fgene.2019.00319 (PMC6465778; doi:10.3389/fgene.2019.00319)

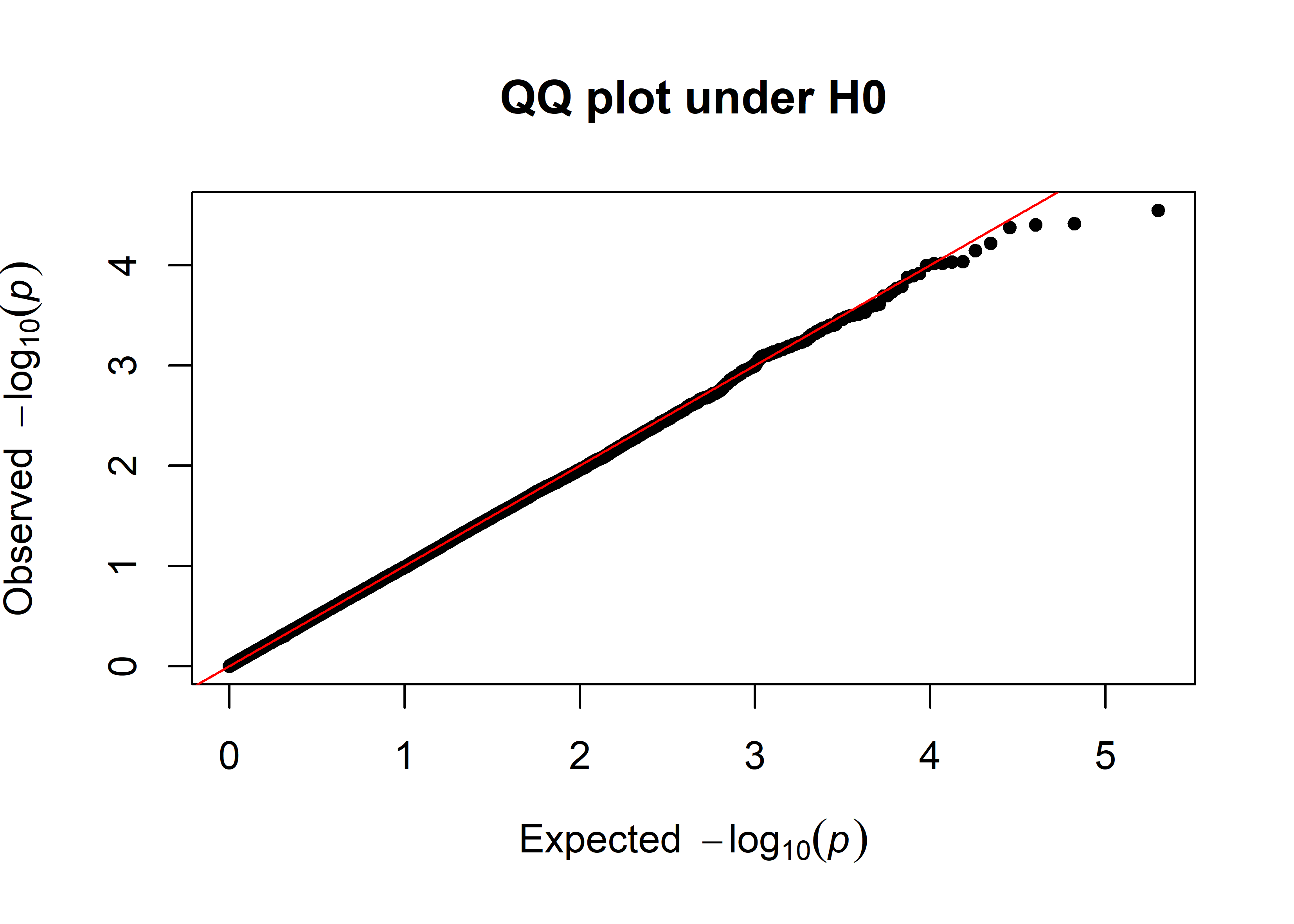

Supplement: FIGURE S1 — QQ plot under H0. [file Image_1.TIFF]

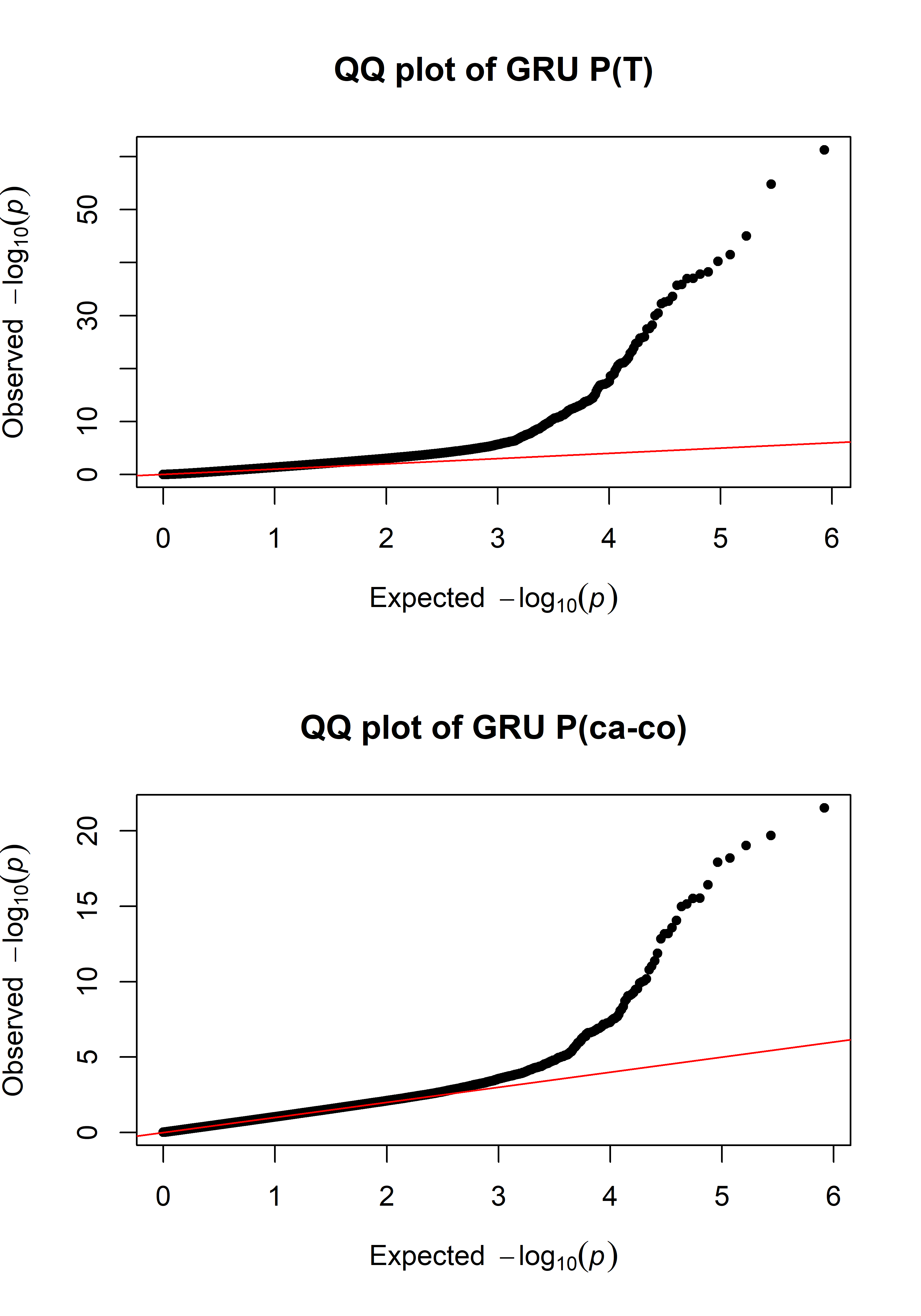

Supplement: FIGURE S2 — QQ plot of P(T) and P(Ca-Co) of GRU Group. [file Image_2.TIFF]

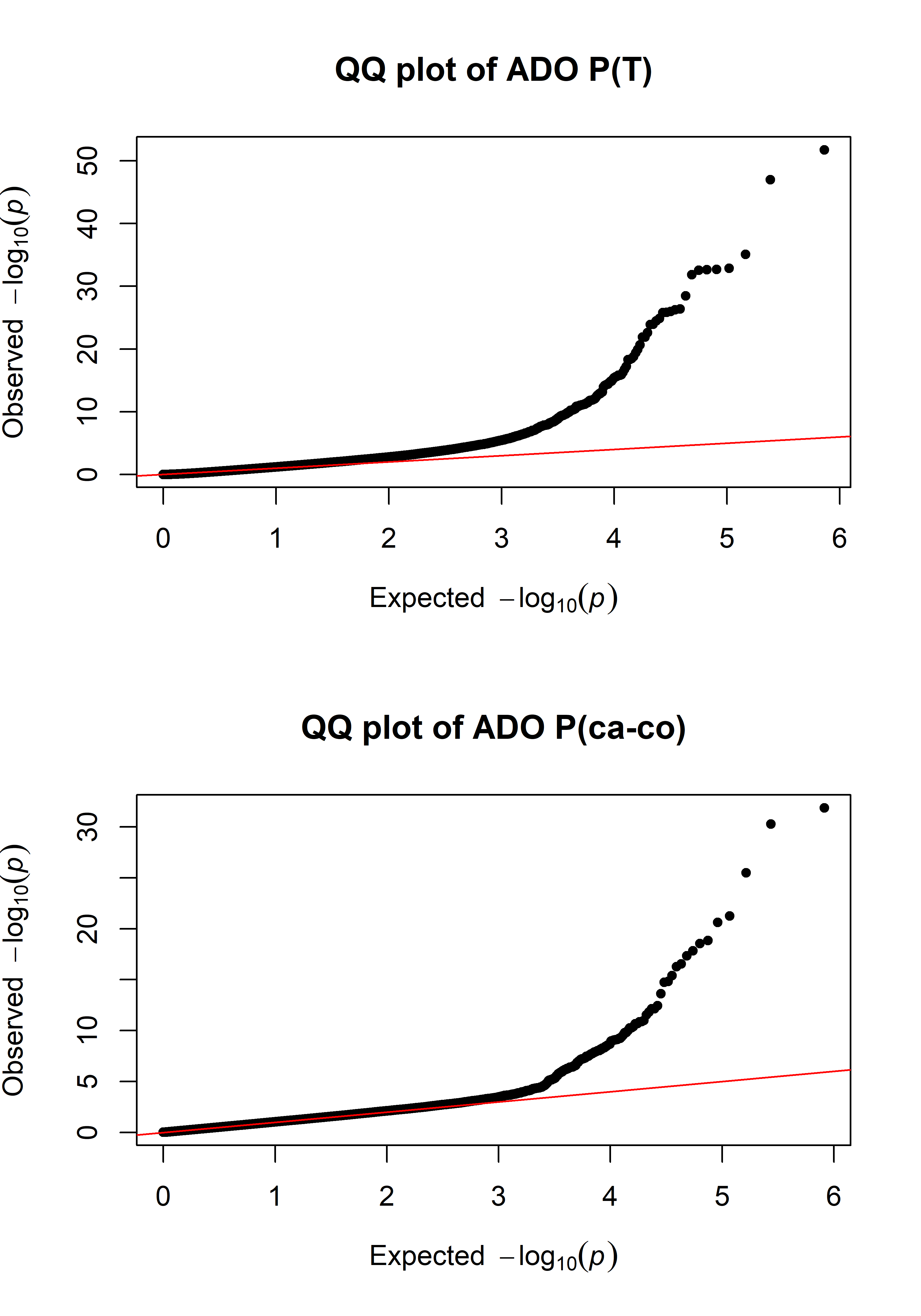

Supplement: FIGURE S3 — QQ plot of P(T) and P(Ca-Co) of ADO Group. [file Image_3.TIFF]

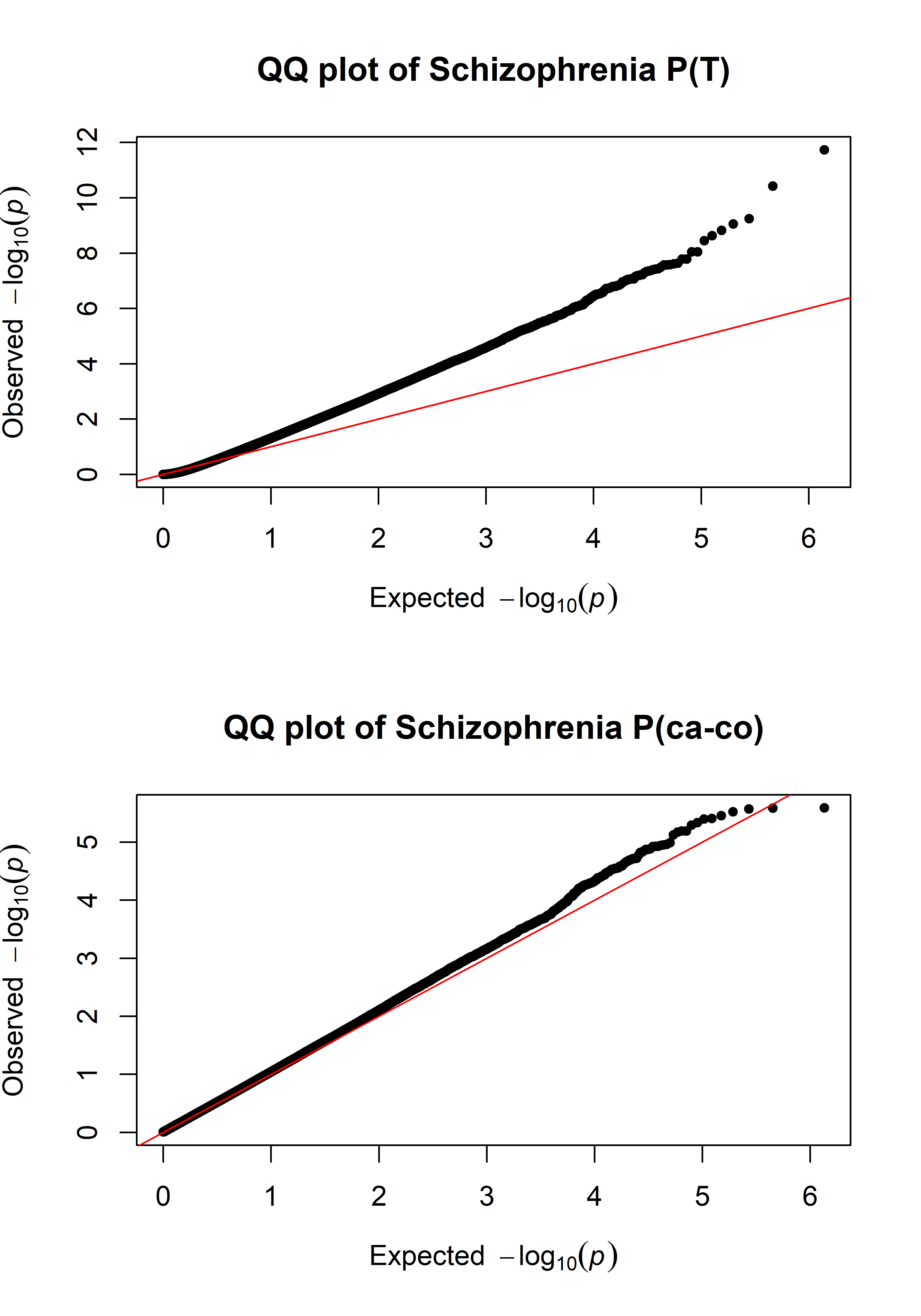

Supplement: FIGURE S4 — QQ plot of P(T) and P(Ca-Co) of Schizophrenia. [file Image_4.TIFF]
